# Combining International Standards to Develop Clinical Decision Support for Parent Smoking Cessation in Pediatrics

**DOI:** 10.2196/75198

**Published:** 2025-11-05

**Authors:** Jeritt G Thayer, Brian P Jenssen, Alexander G Fiks, Dean Karavite, Janani Ramachandran, Shannon Kelleher, Ekaterina Nekrasova, Jeremy E Drehmer, Emara Nabi-Burza, Jonathan P Winickoff, Robert W Grundmeier

**Affiliations:** 1 Department of Biomedical and Health Informatics, Children's Hospital of Philadelphia, Philadelphia, PA, United States; 2 Department of Pediatrics, Perelman School of Medicine, University of Pennsylvania, Philadelphia, PA, United States; 3 Clinical Futures, Children's Hospital of Philadelphia, 2716 South Street, Philadlphia, PA, 19146, United States, 1 267-425-1699; 4 Tobacco Research and Treatment Center, Division of General Academic Pediatrics, Massachusetts General Hospital for Children, Massachusetts, MA, United States

**Keywords:** clinical decision support systems, health information technology, health information standards, health information interoperability, smoking cessation, population health

## Abstract

Smoking is associated with severe health consequences. Secondhand smoke exposure among children increases the risk of sudden infant death syndrome, chronic respiratory diseases such as asthma, and lung cancer in adulthood. For many parents, pediatricians are the primary source of interaction with the health care system. Nevertheless, in pediatric settings, appropriate tobacco treatments are rarely, if ever, provided to parents who smoke. To best address tobacco use among parents, it is ideal to develop scalable solutions that are coordinated across health systems, community partners, and national services within pediatric settings. We describe our experience in developing and implementing a parent tobacco treatment platform within a pediatric institution that leverages multiple international standards to support interoperability, with the overarching goal of providing a model for how such work can be approached. The clinical decision support (CDS) system includes clinician- and patient-facing components, connects parents to 3 different treatment options (nicotine replacement therapy, text-based counseling, and telephonic counseling), and incorporates 3 international standards (Fast Healthcare Interoperability Resources [FHIR], SMART on FHIR, and CDS Hooks). FHIR is used across all components. SMART on FHIR is limited to the clinician-facing tool, and CDS Hooks is used in the patient-facing portion. While health care interoperability standards supported a significant portion of the overall system, nonstandard technologies and enhancements of existing standards were also required. Furthermore, no connections with community partners could use existing interoperability standards. Over one year, the CDS was used in 194,946 visits, identified 7847 parents who smoked, and connected 2954 parents to 6320 distinct treatment services, a significant improvement compared to prior efforts. Our project demonstrates that building CDS systems using international standards, such as SMART on FHIR, FHIR, and CDS Hooks, is possible, but challenges remain. Limits in the CDS Hooks standard to support common workflows and a lack of communication standards used by third parties outside the health care system represent areas for future work. To support these requirements, additional electronic health record–specific records and communication mechanisms are required.

## Introduction

Smoking causes severe health consequences, and there is no safe level of secondhand smoke (SHS) exposure [1,2]. SHS exposure among children increases the risk of sudden infant death syndrome, chronic respiratory diseases such as asthma, and lung cancer in adulthood [2]. Over 40% of children in the United States are regularly exposed to SHS, most often by a parent [3]. When a parent quits smoking, they significantly lower their chances of developing lung and other related cancers and lengthen their life expectancy [4]. Parental smoking cessation eliminates the majority of children’s SHS exposure and decreases the odds that their children will become tobacco users themselves [5-7]. Pediatricians are well positioned to help parents quit smoking by implementing interventions that connect parents with cost-effective, evidence-based tobacco cessation treatments [8,9]. Furthermore, for low-income households, where the smoking rate can be 2 times the general population level, pediatricians are often parents’ primary source of interaction with the health care system [10,11]. Thus, integrating tobacco cessation treatment within pediatric care holds great promise for mitigating the harm of tobacco within families.

While pediatric health care systems are uniquely positioned to help parents and household members (henceforth referred to as parents) quit smoking, evidence-based treatments are significantly underused [12]. Cost-effective cessation strategies exist, especially when behavioral and pharmacological interventions are combined (eg, nicotine replacement therapy [NRT] plus the Quitline) [13]. Compared to controls, behavioral interventions, such as telephone- or text-messaging–based counseling, and NRTs significantly increase smoking cessation rates and can easily be disseminated [14,15]. The 2021 US Preventive Services Task Force, Healthy People 2030, the Department of Health and Human Services, and the surgeon general all strongly recommend that clinicians ask all adults about tobacco use and provide behavioral interventions and pharmacotherapy for cessation [13,16-18]. To best address tobacco use among parents, it is ideal to develop scalable solutions that are coordinated across health systems, community partners, and national services.

Fortunately, advances in health interoperability standards, such as Fast Healthcare Interoperability Resources (FHIR), SMART on FHIR (hereafter referred to as SMART), and CDS Hooks, have created opportunities to more easily support these efforts [19-21]. FHIR is a health information exchange standard that allows clients (eg, clinical decision support [CDS] services) to obtain granular access to data, such as encounters, allergies, or medications, using a standardized application programming interface (API). SMART is a standard that allows authorized apps to be opened directly from an electronic health record (EHR) or patient portal so that clinicians and patients can seamlessly access external apps within their workflow. CDS Hooks is an event-driven framework in which specific actions in the clinical workflow, such as ordering a medication and trigger messages, known as “hooks,” are sent to subscribing services. These systems can then return recommendations as a “card” to the user directly within their EHR workflow. Systems built on these standards, known as service-oriented systems, are separate from the host system (eg, EHR) but connected using a standard API [22]. This allows for a modular, scalable approach that can extend care beyond the individual patient.

Several researchers have already begun to explore using these standards for developing CDS systems. Investigators in one health system demonstrated the utility of the SMART framework within the EHR using both clinician- and patient-facing apps to address clinical needs such as patient-specific medication instructions [23]. Dolin et al [24] developed a prototype pharmacogenomics CDS service that interfaced with a commercial EHR using the CDS Hooks standard, and Jung et al [25] used CDS Hooks to implement a national service for drug allergy interaction checking. Other researchers have sought to combine standards within the same system. Theiss et al [26] developed a prototype app that combined several health information technology standards in a nonproduction setting, and Morgan et al [27] combined contextual CDS Hooks prompts to direct clinicians to SMART apps within their hospital’s emergency department.

These studies highlighted the utility of each standard; however, the apps were designed mainly to support clinicians, not patients or families, and were deployed in nonpediatric settings. Lastly, while some use cases have connected to external systems (eg, Value Set Authority Center or RXNorm) to retrieve information, the apps did not interface with community health care partners. In this viewpoint, we describe our experience in developing and implementing a parent tobacco treatment platform (PTTP) within a pediatric institution that leverages multiple international standards to support interoperability, with the overarching goal of providing a model for how such work can be approached. We highlight three key areas where critical decisions shaped the design and trajectory of the system: (1) international standards, (2) community partnerships, and (3) family-centered care. By sharing these lessons, we aim to guide other health IT developers, physicians, health care leadership, and standards development organizations in designing, implementing, and scaling CDS interventions.

## How We Designed the PTTP

### Project Setting

The PTTP was developed as part of a National Institutes of Health funded research initiative focused on connecting the parents (including caregivers and other household members) of our patients with smoking cessation services within the Children’s Hospital of Philadelphia (CHOP) Care Network and Pediatric Research Consortium [28], which includes a combination of suburban, urban, and semirural practices that use a common EHR (Epic Systems Inc). The tool was initially deployed in 5 clinics and has since expanded across all CHOP’s primary care and pulmonary medicine networks (35 sites). Development (including user analysis and design) of the CDS system occurred between January 2020 and November 2023. To support this work, a multidisciplinary team was formed that consisted of physicians, a software engineer and EHR-integration expert, a human-computer interaction specialist, and project managers. The project was approved by the CHOP Institutional Review Board (IRB 20‐018146).

### System Overview

**Figure 1.**
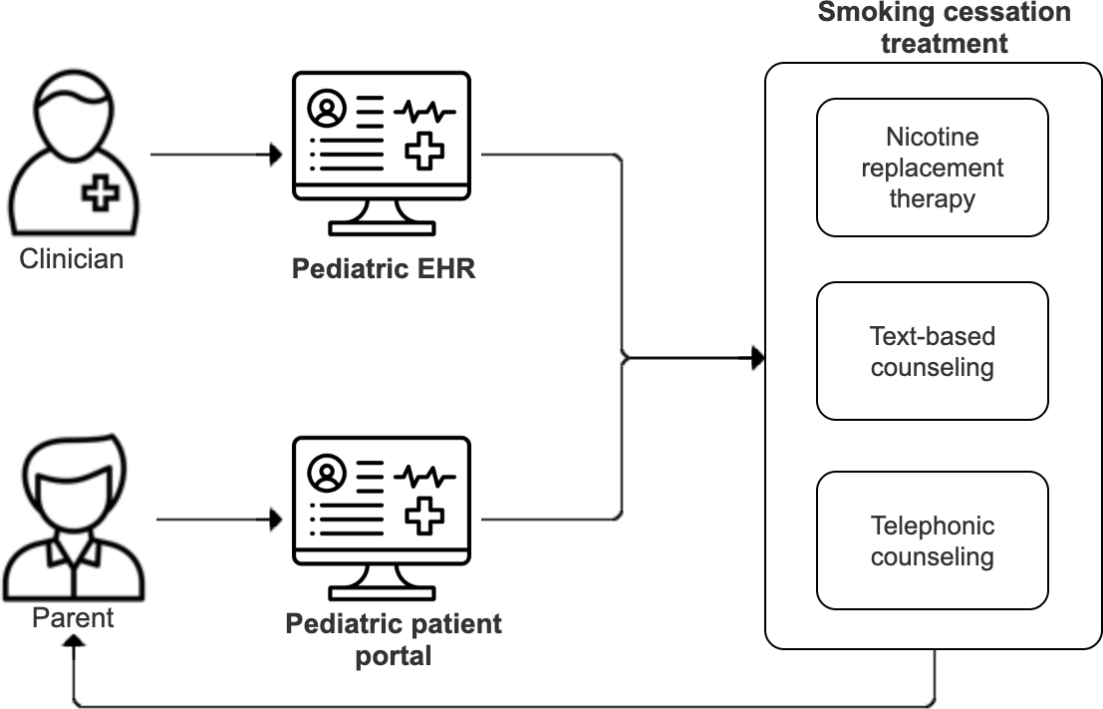
Conceptual framework for connecting parents to tobacco cessation treatment from the pediatric chart. EHR: electronic health record.

On the basis of a rigorous human-centered design evaluation, which consisted of interviews with clinicians, parents, and front-desk staff, formative testing of prototypes, and iterative feedback sessions, we identified that the system needed to support 2 high-level workflows (Figure 1) [29,30].

**Figure 2.**
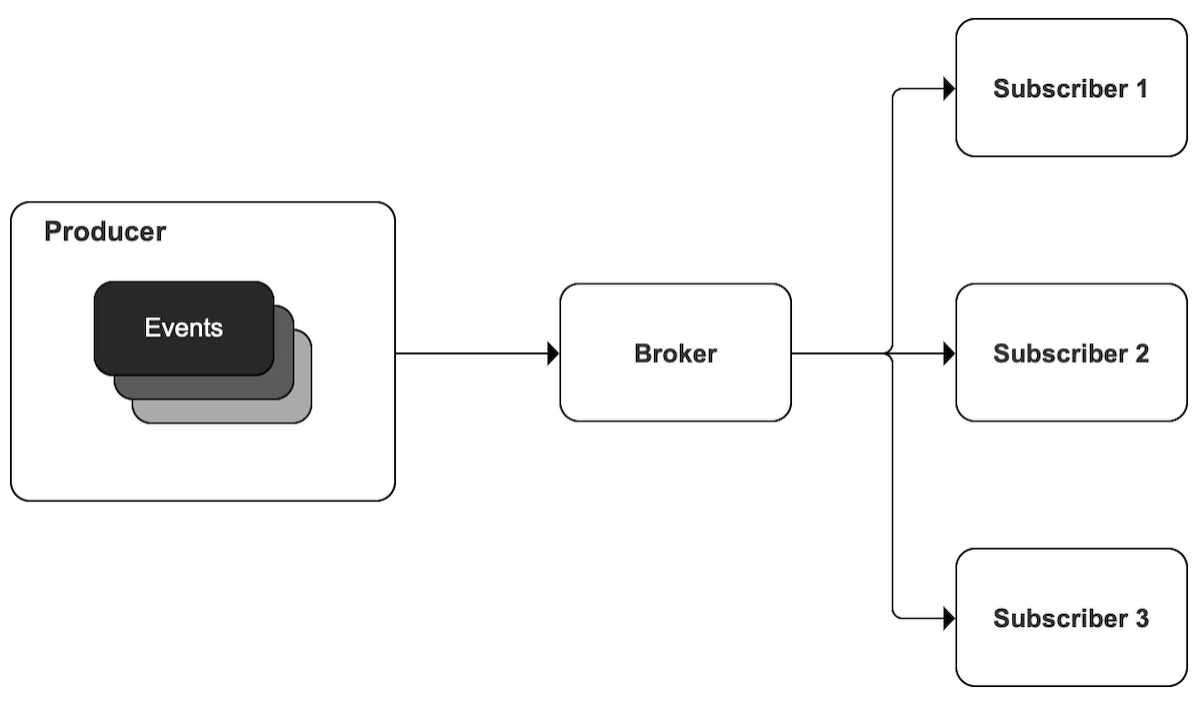
Event-driven architecture.

First, the system needed to screen, identify, and automatically connect parents to the requested treatment options. Although some health care providers (HCP) already routinely ask parents about their smoking status, our team found that the process is inconsistent. Furthermore, parents felt more comfortable disclosing their smoking status through a survey that used nonjudgmental language [29]. Additionally, to engage parents during a potential activation window, where they are energized to engage in treatment, all treatment connections needed to be processed immediately upon user responses. From these priorities, we hypothesized that an event-driven architecture, responding to previsit questionnaires in the patient portal, would meet the needs of our project. This architecture includes the following high-level layers: (1) producer (source of events), (2) broker (sender of events), and (3) subscriber (listener of events; Figure 2) [31]. Questionnaires can be completed up to 7 days ahead of the visit within the patient’s personal health portal (MyChart; Epic System), during check-in, or by the HCP during the visit. The questionnaire is configurable within our vendor EHR and includes questions about smoking use; if the respondent indicates that they smoke, it provides a motivational message to encourage treatment engagement and three evidence-based treatment options: (1) NRT, (2) Quitline enrollment, and (3) SmokefreeTXT enrollment. The Quitline is a free, state-specific, telephone-based smoking cessation service that provides counseling, referrals to other programs, and access to free medication. SmokefreeTXT is a free mobile text messaging service offered by the National Cancer Institute that provides daily tips and 24-hour support for quitting smoking. Treatment connections are processed immediately upon completion of the questionnaire.

**Figure 3.**
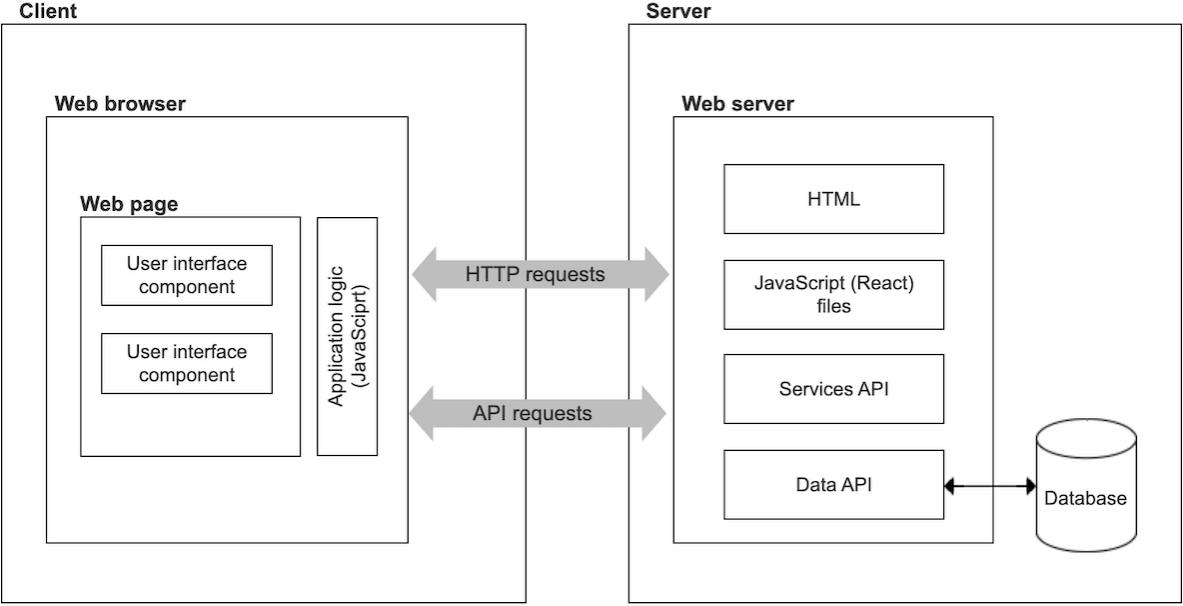
Client-side rendering architecture. API: application programming interface.

Second, the system should incorporate a component embedded within the clinician workflow in the EHR. HCPs were interested in understanding the smoking status of the household, given its impact on the child. To reduce the burden on HCPs, who have to juggle many competing priorities in a limited amount of time, the clinician-facing component needed to not only summarize smoking use in the household but also quickly allow HCPs to connect parents to smoking cessation treatment and support other clinical tasks within the EHR, such as documentation and billing. We hypothesized that a client-side rendering architecture would serve the needs of this component [32]. This architecture includes the following layers: (1) presentation (eg, the user-facing app), (2) application (eg, clinical logic), (3) service (eg, connection to database and treatment partners), and (4) database (storage of app data; Figure 3).

## Interoperability Standards Evaluation

We mapped the architectural needs of our system to interoperability standards across all versions supported by Health Level 7 (HL7) International (draft standard for trial use, standard for trial use, and release). To reduce potential dissemination barriers of our system to other organizations, we prioritized standards based on modern internet protocols (eg, FHIR) and limited them to only those well supported by our commercial EHR vendor. Refer to Table 1 for the list of standards that were evaluated.

**Table 1.** Interoperability standards reviewed.

Standard	Description	Component
CDS Hooks [21]	An event-driven architecture, where events in the EHR^a^ (known as hooks) trigger messages to subscribers. Currently an HL7^b^ standard for trial use, it uses FHIR^c^ for the data model standard, and OAuth 2.0 and OpenID Connect for authorization and authentication.	Parent
Direct messaging [33]	Method of securely exchanging information to trusted partners. Commonly used with Simple Mail Transfer Protocol and the Continuity of Care Document as a method for exchanging clinical information. Used by some EHRs to connect patients to telephone-based counseling services.	Parent
Clinical Quality Language [34]	A standard for representing clinical logic to enable sharing across institutions. The language is both human- and machine-readable, and common use cases include quality measures and decision support.	Parent and clinician
FHIR [19]	Health information exchange standard that takes advantage of popular internet protocols and data formats. Increasing in popularity among health care developers and now on release 6.	Parent and clinician
SMART [20]	Standard to integrate external apps into health information systems such as the EHR. Uses OAuth 2.0 and OpenID Connect for authorization and authentication and FHIR for information exchange standard.	Clinician
Web messaging [35]	HL7 standard for supporting tighter integration between the host system and embedded SMART app. Relies on HTML5’s web-messaging protocol to support communication between systems. While not directly supported in our EHR, our EHR vendor provides a similar web-messaging framework, which is included as part of our evaluation.	Clinician

a EHR: electronic health record.

b HL7: Health Level 7.

c FHIR: Fast Healthcare Interoperability Resources.

While HL7 version 2 messaging is the most common event-driven standard used in health care, its implementation often uses a transmission control protocol, which is at a lower level of the Open Systems Interconnection model compared to more modern standards, such as FHIR [36]. As a result, it is generally not a preferred health information exchange standard for new developments. Evaluations occurred at the following technology layers, where standards were available: (1) EHR-integration framework (eg, SMART, CDS Hooks, and web messaging), (2) data model (eg, FHIR), (3) logic (eg, clinical quality language [CQL]), and (4) community information exchange (eg, direct).

## Interoperability Standards Selection

**Figure 4.**
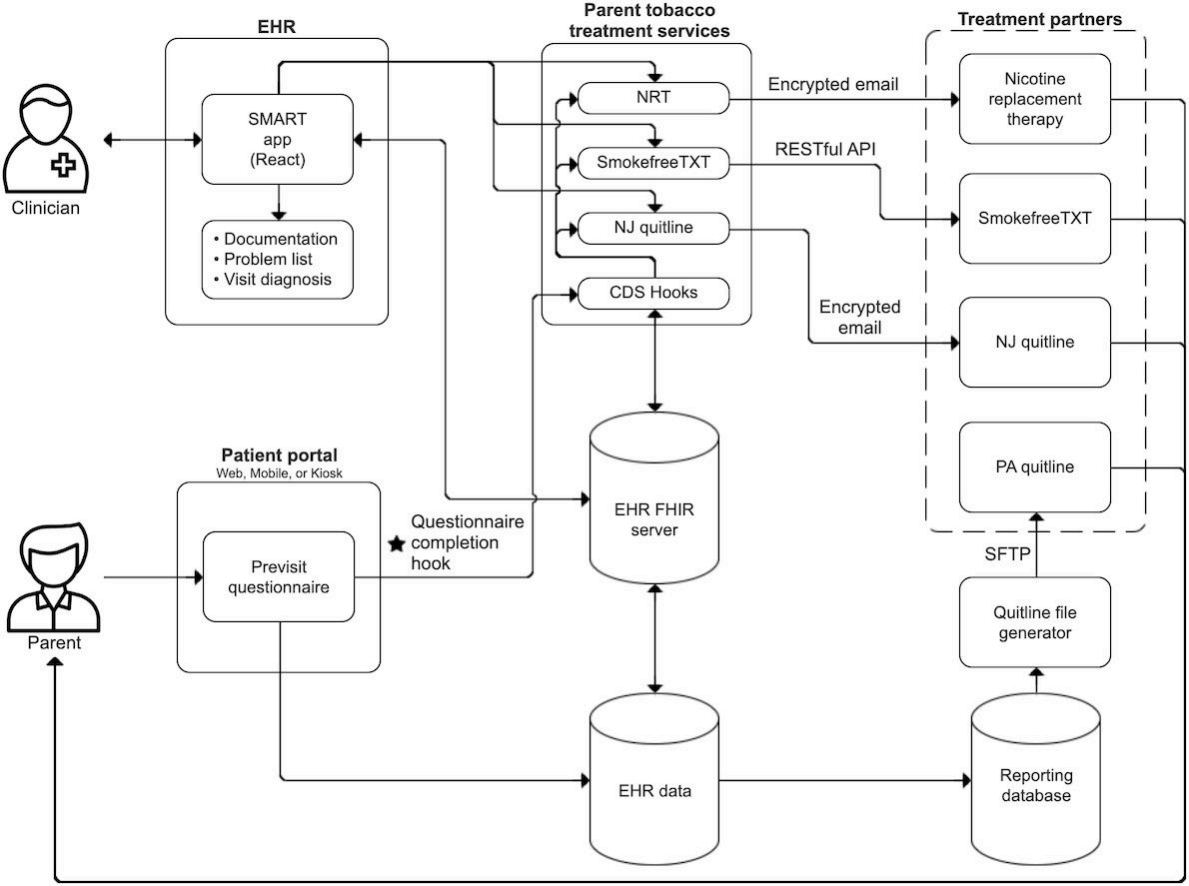
Parent tobacco treatment platform. API: application programming interface; CDS: clinical decision support; EHR: electronic health record; FHIR: Fast Healthcare Interoperability Resources; NRT: nicotine replacement therapy; PA: Pennsylvania; SFTP: secure file transfer protocol.

The smoking cessation system (Multimedia Appendix 1) has 2 high-level components, a parent-facing and a clinician-facing system, with services shared between them (Figure 4). Both components are described below, with a separate section dedicated to shared services. Table 2 provides an overview of the use of standards across each component.

**Table 2.** Feature type per component.

Component, feature type, and feature	Notes
Clinician	
Standards	
SMART app launch	Used per specification
FHIR^a^	Used per specification
SMART Web Messaging	Not available; used vendor provided web-messaging framework
Vendor	
Documentation templates	Used to automate clinical documentation
Order sets	Used to automate clinical documentation
Web messaging	Used to “listen” to clinical events, such as when a patient’s problem list was updated
Patient	
Standards	
CDS Hooks	Partially used; required enhanced trigger set provided by our vendor EHR^b^
FHIR	Used per specification
CQL^c^	Not used
Vendor	
Alert framework	Extends the “hooks” available as part of CDS Hooks specification

a FHIR: Fast Healthcare Interoperability Resources.

b EHR: electronic health record.

c CQL: clinical quality language.

### Patient-Facing Component

We first evaluated technologies that would meet the needs of the parent-facing CDS, which was delivered through a previsit questionnaire. Given the existing support for the direct messaging protocol within our EHR and the Pennsylvania Quitline, we initially attempted to facilitate connections using this method. However, as CHOP is a pediatric hospital and does not maintain charts for adults, the Direct protocol did not readily support transmitting information about the parent through the child’s chart. Additionally, other treatment partners did not support the direct protocol. Therefore, a separate service was required to create a connection between the parent and our community treatment partners.

We then evaluated the CDS Hooks standard. While CDS Hooks services are traditionally used to deliver information (eg, recommendations) in the form of a card within a user’s (eg, clinician) workflow, we were interested in using the standard to perform asynchronous tasks. Unfortunately, the current version of the standard does not support a hook based on the completion of a questionnaire. However, our EHR’s implementation of CDS Hooks provides a richer set of triggers, including the completion of a previsit questionnaire. While the use of widely available standards was preferred, other options would have required significant customization and project-specific code. As a result of this and early demonstrations related to adding a hook for responding to patient-completed questionnaires [37], we chose to move forward with the CDS Hooks. Immediately after submitting the questionnaire, a *Patient_View* hook is immediately triggered from the EHR to our system. The system authenticates the message, evaluates questionnaire responses retrieved via FHIR, and connects parents to the requested treatment options.

After determining the trigger mechanism, we explored CQL as a standards-based method for evaluating the patient’s data to determine whether treatment options were requested. While the CQL rule performed as expected, part of the EHR configuration required to send a CDS Hooks message after completion of a previsit questionnaire included a native EHR rule, which already evaluated questionnaire responses. Given this and the additional infrastructure required to support the CQL standard, such as the rules engine, which is not readily supported by our EHR, the CQL rule was considered redundant and removed. In its place, a combination of EHR-based rules within the questionnaire configuration and logic statements in the CDS Hooks service was used.

### Clinician-Facing Component

Next, we evaluated standards-based methods for developing and implementing the clinician-facing component. Given the recent federal regulation mandating support for SMART [38], we chose this method to embed this component directly within the HCP’s EHR workflow, requiring no additional clicks to navigate to or open. The app was written in React (JavaScript) and supports multiple HCP tasks within the EHR. For parents who smoke but do not select a treatment during the questionnaire, the app allows HCPs to enroll the parent in treatment if they change their mind. The system also allows HCPs to add SHS to the problem list with a single click and includes a section with prompts on how to discuss tobacco use with parents and treatment information. Furthermore, the app automates several routine tasks, including populating text within the encounter note and after-visit summary and defaulting visit diagnoses in order sets. When a parent accepts treatment and counseling is performed by the HCP, the system also provides information on how to appropriately bill for the visit.

To facilitate a tight integration between the app and the EHR, we took advantage of our EHR vendor’s web-messaging framework that enables navigation to different areas of the chart, such as viewing questionnaire responses. Although similar to SMART Web Messaging, our EHR’s framework also allows embedded apps to “listen” to events originating from the EHR, such as when a patient’s medications or problem list are updated. For example, to ensure that SHS is not added to the patient’s chart multiple times, our system listens for changes to the patient’s problem list and updates itself accordingly.

## Community Partner Connections

Both the parent- and clinician-facing components use a set of shared services to connect parents to smoking cessation treatment. At the time of our project, limited options existed to support information exchange between the EHR and community partners. Unfortunately, due to varying technical resources across our treatment partners, as well as the lack of support for family-centered care within our EHR, standards-based methods for connecting parents from the child’s chart were not possible. As a result, a separate service was required to connect parents to each partner, as detailed in the subsequent sections. All services are secured using the OAuth 2.0 token infrastructure.

### NRT Treatment

Since the smoking cessation system was embedded within the child’s chart, traditional methods to prescribe medication (eg, E-Prescribe) were not available. To connect parents to NRT, we contracted a third-party pharmacy that delivered medication to the parents’ homes. However, as a smaller organization, the pharmacy lacked the resources to develop a process for ingesting parent information using more modern techniques, such as an API. Consequently, we developed a service to send parent information and a system-generated PDF of a prescription using encrypted email.

### Text Message–Based Counseling

We partnered with SmokefreeTXT to provide text-based counseling to parents. SmokefreeTXT is a text message program provided by the National Cancer Institute that provides a web form that individuals can complete to enroll [39]. We initially attempted to integrate this web form directly into our previsit questionnaire, but due to network restrictions within our organization, this was not possible. Additionally, because the questionnaire includes other treatment options beyond SmokefreeTXT, the use of the web form may have required users to enter their information multiple times if they were interested in other treatment options. At the time of our project, no automated mechanism existed to connect individuals to the program, so we engaged directly with the SmokefreeTXT operating partner to request the development of an API. The API initially only accepted the participant’s phone number; however, a primary goal of our project was to remove as many potential barriers to treatment engagement as possible. As a result, to streamline the enrollment process, which required the user to respond with additional information after enrolling (eg, target quit date and current age), a second API was enabled to programmatically update these values, using information already provided in the questionnaire.

### Telephone-Based Counseling

Because CHOP provides care across multiple states, we needed to partner with 2 separate telephone counseling organizations in Pennsylvania and New Jersey, each with different connection requirements. Quitline selection is determined by the location of the participating clinic. The Pennsylvania Quitline supports the use of the Direct Messaging protocol. Unfortunately, as mentioned above, Direct Messaging does not account for the family-centered nature of care and can only readily be used if the connection originates from the patient’s chart. In an attempt to establish real-time connections, we evaluated the development of an API similar to that used for SmokefreeTXT; however, this was not possible at the time due to limited resources. Additionally, the Pennsylvania Quitline had already established a robust infrastructure where enrollments are processed through a daily file exchange using the Secure File Transfer Protocol. To build the file, a script queries our clinical data warehouse to identify the participants who are requested to be enrolled. This file is then retrieved by the Quitline partner, who is given remote access to the directory, and processed by their system. For our New Jersey partner, due to resource constraints, connections are sent using encrypted email in a process similar to that of our pharmacy partner (described above).

## Impact on Family-Centered Care

Parents want their child’s physician to discuss smoking cessation treatment with them [13]. However, in pediatric settings, appropriate tobacco treatments are rarely, if ever, provided to parents who smoke [12]. This is largely due to workflow limitations in the pediatric EHR, especially the inability to account for the family aspect of child health care [40,41]. EHR vendors often develop features for the physician-adult patient dyad. However, as noted above, they provide limited support to connect family relationships, making it challenging to leverage existing technologies to support teamwork across the patient’s entire network (eg, care team and community groups), particularly in pediatric settings [42].

Our system focused directly on the family-centered nature of a child’s care. Therefore, over a 1-year period, our system was used in 194,946 visits and identified 7847 (4.03%) parents who smoked. Of those who smoked, 37.64% (n=2954) selected at least one treatment option, 29.27% (n=2297) selected NRT, 29.46% (n=2312) selected SmokefreeTXT, and 21.8% (n=1711) selected the Quitline (Table 3). Performance of the app was excellent, with 99% of all treatment connections handled through the previsit questionnaire and CDS Hooks service without clinician involvement. The remaining 1% of the connections were facilitated using the clinician-facing tool. Interaction (eg, clicks) within the clinician-facing system was limited, occurring in only 774 (<1%) encounters. Given the low level of interaction with the system by HCPs, routine check-ins with practice management were established to ensure that any issues with usability were identified and corrected. No issues were identified during the project period. The uptime of the system (across all components) was 99.99%, with most outages related to network faults that occurred for less than 1 minute. We identified multiple failures in the web-messaging framework; however, these only limited navigation to different areas of the chart from within the app (eg, viewing the questionnaire) and did not impact parent connections. The median (IQR) response time across all system services was 60 (14,393) milliseconds.

**Table 3.** Questionnaire completion and treatment connection rates.

Metric	Count, n (%)
Questionnaires assigned	267,282 (100)
Questionnaires completed	194,946 (73)
Patient portal	99,063 (51)
Visit check-in kiosk or tablet	94,561 (49)
During visit by clinician	1322 (<1)
Identified person who smokes	7847 (4)
Patient portal	2946 (38)
Visit check-in kiosk or tablet	4836 (62)
During visit by clinician	65 (<1)
Treatment connection	
Any	2954 (38)
NRT^a^	2297 (29)
SmokefreeTXT	2312 (29)
Quitline	1711 (22)

a NRT: nicotine replacement therapy.

## Discussion and Future Directions

By combining multiple international health data standards and reusing app services across system components (parent and clinician facing), we were able to develop the EHR-integrated PTTP. Our system connected parents (including caregivers and other household members) with smoking cessation treatment at scale, serving hundreds of thousands of families across a diverse patient and practice population. This represents a significant increase compared to prior work [43-45]. Furthermore, by developing our system in a modular way, we helped to lower the barriers to implementation in another health system on the same EHR, which is using components of our platform [46]. However, our work also showed that there are limits to using international interoperability standards to support family-centered care in pediatrics.

First, some standards, such as CDS Hooks, are limited in scope and support only a fraction of the potentially actionable events that occur within a health care system. Engaging with patients and families outside of the clinical encounter is a growing area of interest in the health care community [37]. One method of doing this is through previsit questionnaires, which can be completed in the patient’s health portal. Using our EHR vendor’s enhanced set of CDS Hooks triggers, our system was able to immediately respond to parents completing a previsit questionnaire. Compared to adult settings, where only 7.6% of patients received a medication associated with tobacco use treatment [47], our system connected 29% of the parents. Additionally, 99% of these connections occurred automatically, without requiring any work from the visit HCP. While CDS Hooks was originally intended to support more synchronous workflows, such as validating orders at the time of ordering, our team envisions a much larger role for the standard to support asynchronous workflows that do not require input from the health team. This could help organizations across EHR platforms change how they engage with their patients and even alter the dynamics of an individual visit.

Additionally, variability in health care workflows and implementation of standards across EHR vendors creates a need to adapt even standards-adherent systems for implementation in different health systems [46,48,49]. This is especially true for standards that are flexible to institutional workflows, such as SMART, which, depending on the EHR, can integrate into the EHR at different stages of the visit or locations in the chart. Furthermore, health care is a complex adaptive system, and a single workflow may not work across organizations [50]. Therefore, even for EHRs that support SMART, individual implementations may differ across care domains (eg, inpatient vs primary care) or organizations. Given these limitations, future research and implementation efforts should evaluate whether a combination of standards-based and native EHR technologies would increase portability while reducing maintenance.

Second, standards are not widely adopted outside of health care organizations, such as hospitals or clinics. Recent regulations require EHR vendors to support health interoperability technologies for certification with the Office of the National Coordinator for Health Information Technology [38]. However, smaller, health care–adjacent organizations similar to our community partners may not use commercial EHRs and often lack the resources to support integrations using international standards. Additionally, standards supporting EHR integration with community partners, such as the Direct protocol, do not support connections for others within the patient’s network (eg, parents). As a result, our system needed to support 3 different methods of information exchange (eg, encrypted email, API, and secure file transfer protocol), and even those that shared a protocol required different information within the message. To fully realize the potential of our system to support interoperability at the community partner level, additional work is needed to create a standardized method for exchanging information. One promising standard-based method to support this is the FHIR ServiceRequest profile, which has been part of the United States Core Interoperability implementation guide since version 2 was released in April 2022.

Lastly, our work showed that the concept of the EHR as a hub, where the EHR provides the necessary capabilities to support teamwork across the patient’s entire network (eg, care team and community groups), is not well supported, particularly in pediatrics [51]. For example, if a parent has 2 children, ideally, our system would only request information (via the previsit questionnaire) once. However, as patient records rarely contain family-based associations [42], the parent is presented with the questionnaire multiple times, which may result in parental frustration and potentially conflicting information across their children’s charts.

Performance of the PTTP was acceptable, with near-perfect uptime across all components. However, we identified issues that warranted attention, including network failures and communication with our EHR’s web-messaging framework. While a growing body of work has identified how CDS can fail, the focus has largely been on centralized computing models where the data and the computation are on the same machine [52-54]. Architectures taking advantage of newer HL7 standards create new complexities and failure modes that are not recognized with existing frameworks [55]. To ensure the ongoing safety and effectiveness of service-oriented CDS, more research into these failure modes is necessary.

## Conclusions

We found that it is feasible to develop and integrate CDS using a hybrid architecture that combines several health IT standards such as SMART, FHIR, and CDS Hooks. These types of systems are likely to increase over time; however, additional work is necessary to both expand existing standards to integrate with the EHR and streamline information exchange processes for sharing data, when appropriate, with third-party systems about a patient’s care network (eg, parents) whose behaviors impact the patient. Similarly, improved EHR support for linking patient records of family relationships will help to align health data across families.

## Supplementary material

10.2196/75198 Multimedia Appendix 1 Screenshots of the parent tobacco treatment platform.

## Abbreviations

API: application programming interface
CDS: clinical decision support
CHOP: Children’s Hospital of Philadelphia
CQL: clinical quality language
EHR: electronic health record
FHIR: Fast Healthcare Interoperability Resources
HCP: health care provider
HL7: Health Level 7
NRT: nicotine replacement therapy
PTTP: parent tobacco treatment platform
SHS: secondhand smoke

## References

[1] National Center for Chronic Disease Prevention and Health Promotion (US) Office on Smoking and Health (2014). The Health Consequences of Smoking—50 Years of Progress: A Report of the Surgeon General.

[2] (2006). Office on Smoking and Health (US). The Health Consequences of Involuntary Exposure to Tobacco Smoke: A Report of the Surgeon General.

[3] Tsai J, Homa DM, Gentzke AS (2018). Exposure to secondhand smoke among nonsmokers - United States, 1988-2014. MMWR Morb Mortal Wkly Rep.

[4] Jha P, Ramasundarahettige C, Landsman V (2013). 21st-century hazards of smoking and benefits of cessation in the United States. N Engl J Med.

[5] Wilson KM, Klein JD, Blumkin AK, Gottlieb M, Winickoff JP (2011). Tobacco-smoke exposure in children who live in multiunit housing. Pediatrics.

[6] Johansson A, Hermansson G, Ludvigsson J (2004). How should parents protect their children from environmental tobacco-smoke exposure in the home?. Pediatrics.

[7] den Exter Blokland EA, Engels R, Hale 3rd WW, Meeus W, Willemsen MC (2004). Lifetime parental smoking history and cessation and early adolescent smoking behavior. Prev Med.

[8] Jenssen BP, Walley SC, Boykan R (2023). Protecting children and adolescents from tobacco and nicotine. Pediatrics.

[9] Drouin O, Sato R, Drehmer JE (2021). Cost-effectiveness of a smoking cessation intervention for parents in pediatric primary care. JAMA Netw Open.

[10] Cornelius ME, Wang TW, Jamal A, Loretan CG, Neff LJ (2020). Tobacco product use among adults - United States, 2019. MMWR Morb Mortal Wkly Rep.

[11] Ku L, Bruen BK, Steinmetz E, Bysshe T (2016). Medicaid tobacco cessation: big gaps remain in efforts to get smokers to quit. Health Aff (Millwood).

[12] Winickoff JP, Nabi-Burza E, Chang Y (2013). Implementation of a parental tobacco control intervention in pediatric practice. Pediatrics.

[13] U.S. Department of Health and Human Services (2020). Smoking cessation. A report of the surgeon general. U.S. Department of Health and Human Services, Centers for Disease Control and Prevention, National Center for Chronic Disease Prevention and Health Promotion, Office on Smoking and Health.

[14] Matkin W, Ordóñez-Mena JM, Hartmann-Boyce J (2019). Telephone counselling for smoking cessation. Cochrane Database Syst Rev.

[15] Whittaker R, McRobbie H, Bullen C, Rodgers A, Gu Y, Dobson R (2019). Mobile phone text messaging and app-based interventions for smoking cessation. Cochrane Database Syst Rev.

[16] Krist AH, Davidson KW, US Preventive Services Task Force (2021). Interventions for tobacco smoking cessation in adults, including pregnant persons: US Preventive Services Task Force Recommendation Statement. JAMA.

[17] Tobacco use - healthy people 2030. U.S. Department of Health and Human Services.

[18] (2008). Treating tobacco use and dependence: 2008 update. U.S. Department of Health and Human Services.

[19] Index - FHIR v5.0.0. HL7 FHIR Foundation.

[20] Mandel JC, Kreda DA, Mandl KD, Kohane IS, Ramoni RB (2016). SMART on FHIR: a standards-based, interoperable apps platform for electronic health records. J Am Med Inform Assoc.

[21] CDS Hooks.

[22] Wright A, Sittig DF (2008). A four-phase model of the evolution of clinical decision support architectures. Int J Med Inform.

[23] Bloomfield Jr RA, Polo-Wood F, Mandel JC, Mandl KD (2017). Opening the Duke electronic health record to apps: implementing SMART on FHIR. Int J Med Inform.

[24] Dolin RH, Boxwala A, Shalaby J (2018). A pharmacogenomics clinical decision support service based on FHIR and CDS Hooks. Methods Inf Med.

[25] Jung S, Bae S, Seong D, Oh OH, Kim Y, Yi BK (2022). Shared interoperable clinical decision support service for drug-allergy interaction checks: implementation study. JMIR Med Inform.

[26] Thiess H, Del Fiol G, Malone DC (2022). Coordinated use of Health Level 7 standards to support clinical decision support: case study with shared decision making and drug-drug interactions. Int J Med Inform.

[27] Morgan KL, Kukhareva PV, Warner PB (2022). Using CDS Hooks to increase SMART on FHIR app utilization: a cluster-randomized trial. J Am Med Inform Assoc.

[28] Fiks AG, Grundmeier RW, Margolis B (2012). Comparative effectiveness research using the electronic medical record: an emerging area of investigation in pediatric primary care. J Pediatr.

[29] Jenssen BP, Karavite DJ, Kelleher S (2022). Electronic health record-embedded, behavioral science-informed system for smoking cessation for the parents of pediatric patients. Appl Clin Inform.

[30] Jenssen BP, Kelleher S, Karavite DJ (2023). A clinical decision support system for motivational messaging and tobacco cessation treatment for parents: pilot evaluation of use and acceptance. Appl Clin Inform.

[31] Kleppmann M (2017). Designing Data-Intensive Applications: The Big Ideas behind Reliable, Scalable, and Maintainable Systems.

[32] Pavic F, Brkic L Methods of improving and optimizing react web-applications.

[33] Direct project. Assistant Secretary for Technology Policy.

[34] Clinical quality language specification. HL7 CQL.

[35] SMART web messaging. HL7 FHIR.

[36] Namli T, Aluc G, Dogac A (2009). An interoperability test framework for HL7-based systems. IEEE Trans Inf Technol Biomed.

[37] Tschandl P, Rinner C (2023). Evaluating a CDS Hook for FHIR questionnaires in a SMART on FHIR app and an existing dermatological CDS system. Stud Health Technol Inform.

[38] (2020). 21st Century Cures Act: interoperability, information blocking, and the ONC health IT certification program. Federal Register.

[39] Smokefree.

[40] Blythe MJ, Del Beccaro MA, Committee on Adolescence, Council on Clinical and Information Technology (2012). Standards for health information technology to ensure adolescent privacy. Pediatrics.

[41] Webber EC, Brick D, Scibilia JP (2019). Electronic communication of the health record and information with pediatric patients and their guardians. Pediatrics.

[42] Jenssen BP, Thayer J, Nekrasova E, Grundmeier RW, Fiks AG (2022). Innovation in the pediatric electronic health record to realize a more effective platform. Curr Probl Pediatr Adolesc Health Care.

[43] Jenssen BP, Muthu N, Kelly MK (2019). Parent eReferral to tobacco quitline: a pragmatic randomized trial in pediatric primary care. Am J Prev Med.

[44] Jenssen BP, Bryant-Stephens T, Leone FT, Grundmeier RW, Fiks AG (2016). Clinical decision support tool for parental tobacco treatment in primary care. Pediatrics.

[45] Winickoff JP, Nabi-Burza E, Chang Y (2014). Sustainability of a parental tobacco control intervention in pediatric practice. Pediatrics.

[46] Saleh SN, Kim E, Thayer JG (2025). Sharing a hybrid electronic health record + fast healthcare interoperability resources clinical decision support across health systems: automating smoking cessation for pediatric caregivers. Appl Clin Inform.

[47] Jamal A, Dube SR, Malarcher AM, Shaw L, Engstrom MC, Centers for Disease Control and Prevention (CDC) (2012). Tobacco use screening and counseling during physician office visits among adults--National Ambulatory Medical Care Survey and National Health Interview Survey, United States, 2005-2009. MMWR Suppl.

[48] Dixon BE, Simonaitis L, Goldberg HS (2013). A pilot study of distributed knowledge management and clinical decision support in the cloud. Artif Intell Med.

[49] Wright A, Sittig DF, Ash JS (2015). Lessons learned from implementing service-oriented clinical decision support at four sites: a qualitative study. Int J Med Inform.

[50] Sittig DF, Singh H (2010). A new sociotechnical model for studying health information technology in complex adaptive healthcare systems. Qual Saf Health Care.

[51] Wasserman RC, Fiks AG (2021). The future(s) of pediatric primary care. Acad Pediatr.

[52] Wright A, Hickman TT, McEvoy D (2016). Analysis of clinical decision support system malfunctions: a case series and survey. J Am Med Inform Assoc.

[53] Kassakian SZ, Yackel TR, Gorman PN, Dorr DA (2017). Clinical decisions support malfunctions in a commercial electronic health record. Appl Clin Inform.

[54] Stone EG (2018). Unintended adverse consequences of a clinical decision support system: two cases. J Am Med Inform Assoc.

[55] Thayer JG, Franklin A, Miller JM, Grundmeier RW, Rogith D, Wright A (2024). A scoping review of rule-based clinical decision support malfunctions. J Am Med Inform Assoc.

