# Supplementary material for: Combining International Standards to Develop Clinical Decision Support for Parent Smoking Cessation in Pediatrics
Source: J Med Internet Res. 2025 Nov 5;27:e75198. doi: 10.2196/75198 (PMC12588596; doi:10.2196/75198)
Supplement: Multimedia Appendix 1 [file jmir-v27-e75198-s001.pptx]

## Slide 1
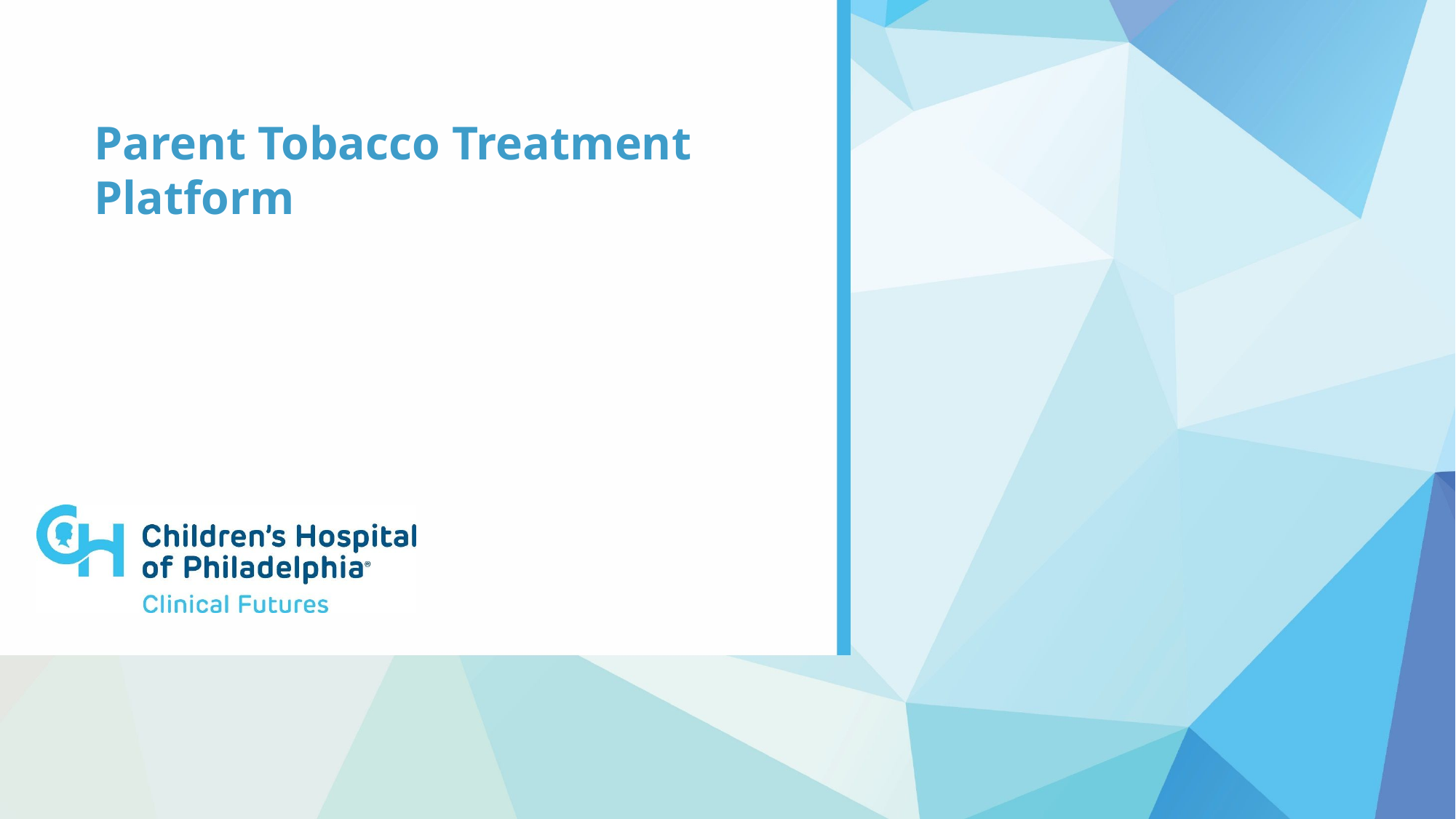

# Parent Tobacco Treatment Platform

## Slide 2
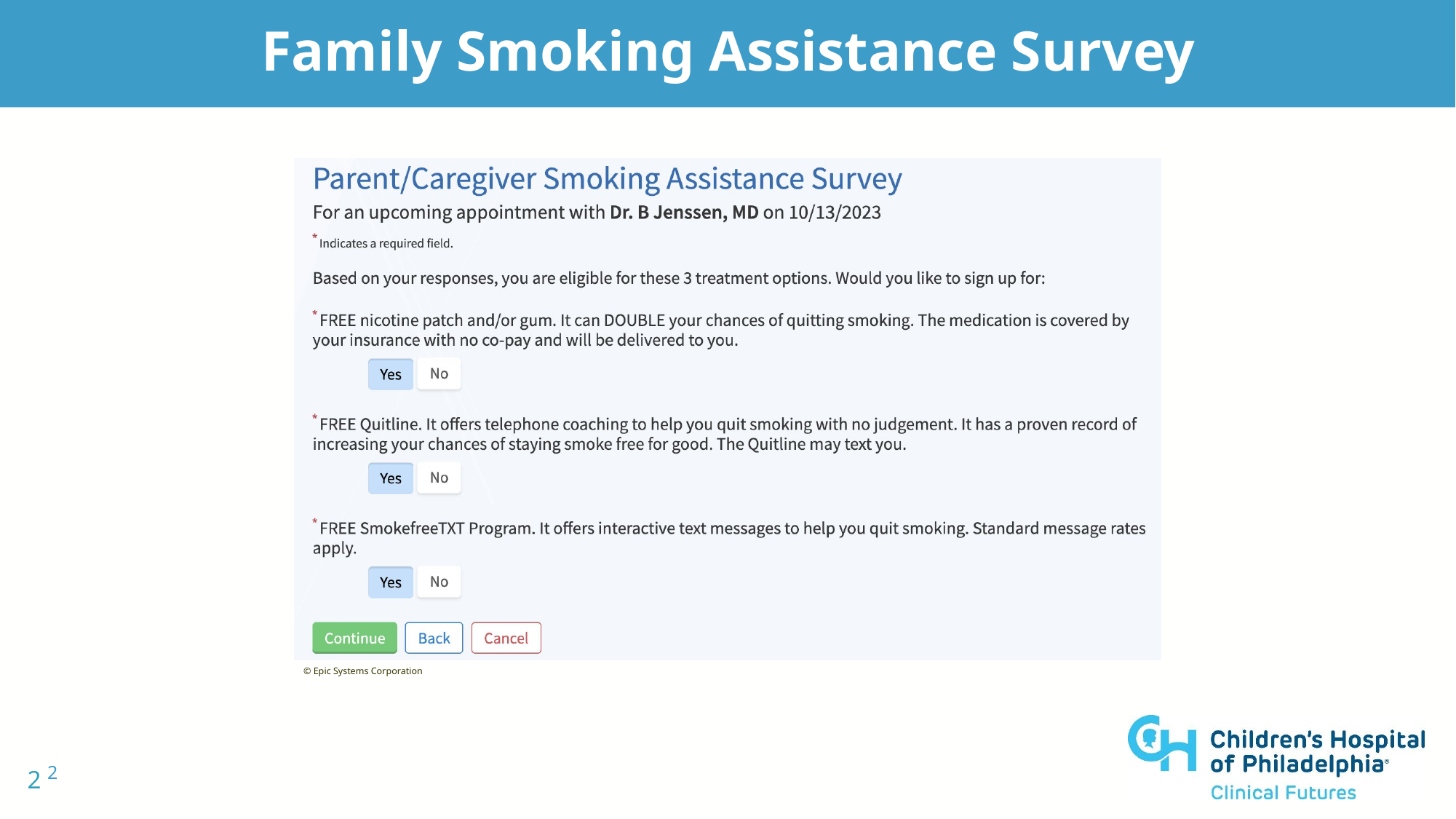

# Family Smoking Assistance Survey
© Epic Systems Corporation
2

## Slide 3
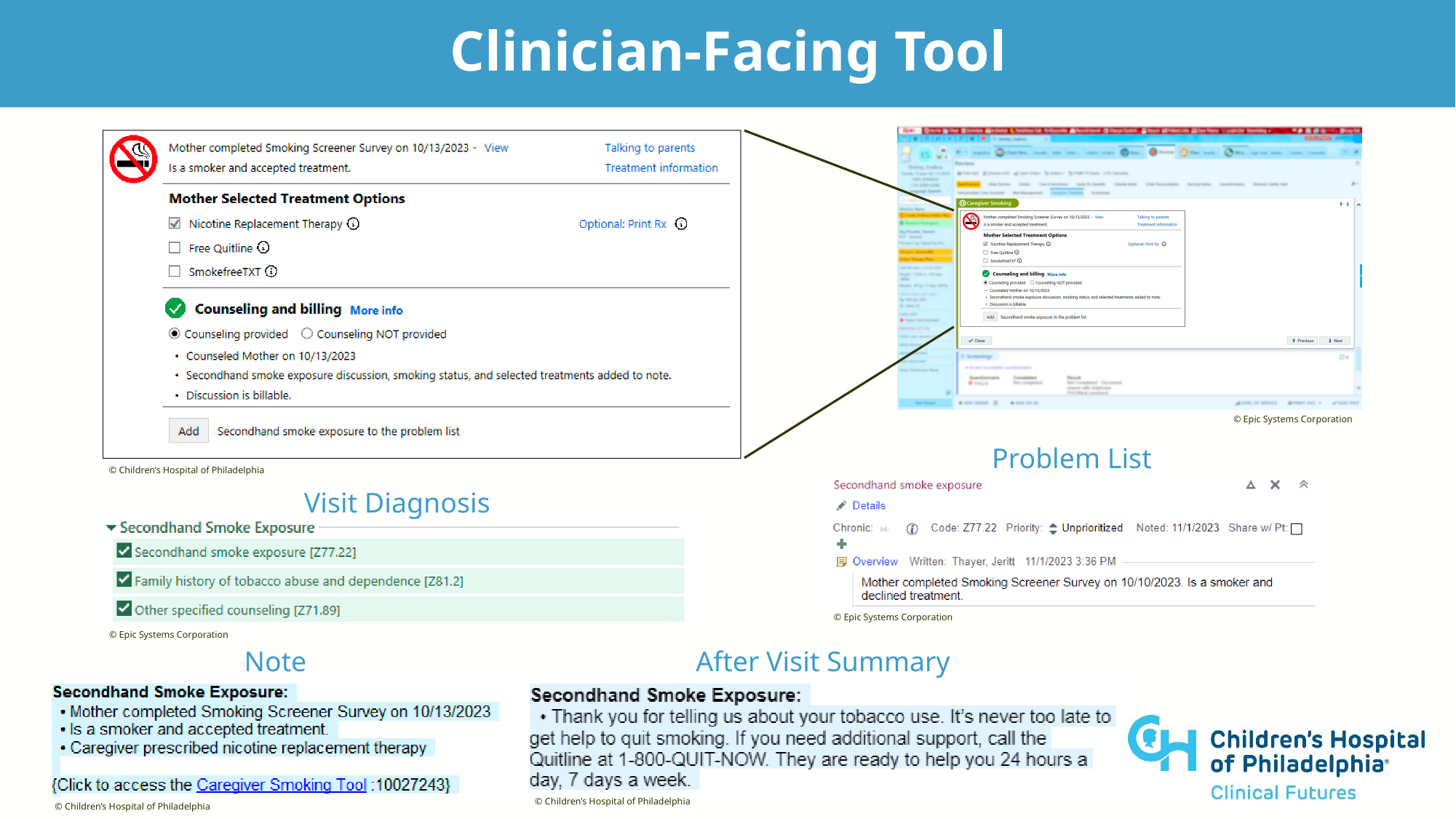

# Clinician-Facing Tool
© Epic Systems Corporation
Problem List
© Children’s Hospital of Philadelphia
Visit Diagnosis
© Epic Systems Corporation
© Epic Systems Corporation
Note
After Visit Summary
© Children’s Hospital of Philadelphia
© Children’s Hospital of Philadelphia
